# Simulation Study on How Input Data Affects Time-Series Classification Model Results

**DOI:** 10.3390/e27060624

**Published:** 2025-06-12

**Authors:** Maria Sadowska, Krzysztof Gajowniczek

**Affiliations:** Institute of Information Technology, Warsaw University of Life Sciences-SGGW, 02-787 Warszawa, Poland

**Keywords:** time series, classification, synthetic data

## Abstract

This paper discusses the results of a study investigating how input data characteristics affect the performance of time-series classification models. In this experiment, we used 82 synthetically generated time-series datasets, created based on predefined functions with added noise. These datasets varied in structure, including differences in the number of classes and noise levels, while maintaining a consistent length and total number of observations. This design allowed us to systematically assess the influence of dataset characteristics on classification outcomes. Seven classification models were evaluated and their performance was compared using accuracy metrics, training time and memory requirements. According to the evaluation, the CNN Classifier achieved the best results, demonstrating the highest robustness to an increasing number of classes and noise. In contrast, the least effective model was the Catch22 Classifier. Overall, the performed research leads to the conclusion that as the number of classes and the level of noise in the data increase, all classification models become less effective, achieving lower accuracy metrics.

## 1. Introduction

The recent enhancement of data accessibility across a wide range of research areas has led to significant developments in time-series processing. This impact is observable in various domains, including not only technology sectors but also medical fields (such as radiomics [1]), astronomy [2], stock markets [3], and energy sectors [4]. Each of these fields has real-life implications for the advancement of their respective sectors, highlighting how time-series analysis has become one of the most challenging problems in today’s technological research landscape. time-series classification has become a crucial part of multiple research problems with the increased availability of temporal data and the high demand stemming from other fields. Its importance is compounded by the significant computational power required for data processing due to their large volume. Therefore, the analysis of classification models in this field is essential in order to handle the data in the most efficient and economical manner.

The main aim of this research paper is to compare the performance of time-series classification models based on the input data. One of the key challenges in time-series classification is selecting an appropriate model that effectively processes temporal data while maintaining computational efficiency. Although many studies explore classification techniques, there is a noticeable gap in research focusing on how different models respond to variations in input data characteristics under controlled conditions. Understanding these differences is crucial for optimizing model selection based on dataset properties. The data used in this study were simulated to enable the most accurate assessment, taking into account specific assumptions about dataset differences that would be nearly impossible to control with real-life data. By using synthetic data, we were able to control factors such as the number of classes, noise distribution, and data contamination, allowing for a comprehensive comparison of the classification models and their response behavior to the input data. Throughout all the considered datasets, the total number of observations remained constant, and the number of observations in each class was balanced to focus on the overall model effectiveness.

Although our study does not employ entropy-based metrics explicitly, it systematically simulates time-series datasets of increasing informational entropy—through variations in class cardinality and noise level—to observe how model performance degrades as data disorder grows. Many of the classification algorithms that were evaluated internally rely on entropy or related impurity measures in their splitting criteria. Therefore, the observed decline in classification accuracy under higher noise and class complexity can be viewed as a manifestation of entropy-driven decision boundaries becoming less distinct in more disordered data. This work thus provides empirical insight into model robustness under entropy-like conditions and lays the groundwork for future extensions that incorporate formal entropy measures into time-series classification analyses.

This study presents the effectiveness of selected models for time-series classification based on different input data. It compares classification results using selected metrics, computational efficiency, and overall performance. Additionally, within the scope of this research paper, all results were reviewed based on the characteristics of the datasets, including components such as the number of classes and data quality. Thus, the research provides a comprehensive analysis of models representing diverse groups, selected based on processing power limitations. The models considered below demonstrated optimal computational efficiency during the experiment, serving as a benchmark for other examinations. A limitation of this study is the use of simulated data, which, while allowing controlled comparisons, may not fully capture the complexity of real-world datasets. Moreover our experiments were conducted using the *aeon* library [5], which provides a wide range of time-series classification models implemented in a standardized and efficient manner. To maintain consistency and avoid introducing external factors or implementation variability, we limited our comparison to models available within this framework.

Our contribution includes:

- the generation of synthetic time-series datasets with controlled characteristics, such as the number of classes, level of noise, and data contamination.
- the comparison of selected time-series classification models based on the generated datasets, with focus on their accuracy and efficiency.
- the analysis of models’ performance in response to changes in input data characteristics, including the number of classes and noise, offering the insights into how different dataset characteristics influence model performance.

The study found that model performance was higher when a smaller number of classes was used, especially under low-noise conditions. As the number of classes increased, maintaining performance required reducing the noise in the data, particularly by lowering its standard deviation. Among the models evaluated, the CNN (Convolutional Neural Network) [6] classifier achieved the highest median performance with statistically significant results and demonstrated the greatest robustness to variations in class count and noise levels. While the ROCKET (RandOm Convolutional KErnel Transform) [7] classifier was the most efficient in terms of training time, the CNN classifier required the longest training duration. In terms of memory usage, the Catch22 (CAnonical Time series CHaracteristics) [8] classifier had the lowest memory footprint despite its lower overall performance.

This paper is structured as follows: Section 2 presents the review of literature, Section 3 outlines the classification models used in the study, Section 4 describes the methodology used for synthetic dataset generation, Section 5 discusses the results of the conducted experiment, and Section 6 concludes the key findings and future directions for this research area.

## 2. Literature Review

In recent years, as time-series data have become more prevalent, numerous research papers have focused on their processing. A significant number of studies has examined the performance of classification models. While most research has aimed at evaluating algorithm effectiveness, only a few studies have explored the use of synthetic datasets to ensure more controlled and reliable comparisons. Although many papers discuss data simulation as a method of creating a more controlled experimental environment, its application in time-series classification remains relatively limited as most of the researchers have focused on real-life data.

The problem of model comparison has been addressed from various perspectives in different studies. Researchers have evaluated algorithms using diverse approaches, often considering computational power to examine how different datasets affect the results. Dhariyal et al. [9] compared tabular machine-learning models with models from the ROCKET family, assessing them with accuracy metrics and the required computational power, measured in minutes. Furthermore, Middlehurst et al. [10] employed a similar approach when comparing datasets, but their research involved training a broader range of models. They compared models within each subset based on the algorithm and then across the subsets. Jiang [11] took a more comprehensive approach, using a wider range of metrics, including accuracy, win rate, average ranks, critical difference diagrams, and pairwise comparisons to compare models in a holistic manner.

The issue of data simulation has been explored by a number of scholars, who have gone beyond generating data for time-series classification. In fact, this is only one of its many applications. Most studies on time-series data generation focus on complex algorithms designed to mimic real-world data, ensuring that distributions and key characteristics are preserved to make the synthetic data as realistic as possible. Numerous models have been developed for this purpose, including TimePFN, which operates based on Gaussian process kernels and linear coregionalization (Taga et al. [12]); RTSGAN (Pei et al. [13]) and TimeLDM (Qian et al. [14]), both utilizing autoencoders; and GuidedDiffTime, which is a set of generative methods proposed by Coletta et al. [15]. However, simpler data generation methods can also be found in the literature, often used for better understanding the effectiveness of different models. Such approaches have been described by Vergara [16] and Sikder et al. [17].

While this study focuses on classification, it is worth noting that related tasks in time-series analysis, such as imputation, forecasting, or anomaly detection, face many of the same challenges. These include dealing with missing data, identifying long-term dependencies, and extracting meaningful patterns from noisy signals. In the context of imputation, recent work has explored the use of boundary-aware diffusion models to reconstruct incomplete sequences more effectively [18]. Forecasting, on the other hand, has seen progress through transformer-based architectures, including decoder-only designs [19]. Anomaly detection also increasingly builds on imputation techniques, combining them with diffusion mechanisms to better detect subtle irregularities in time series [20]. Some approaches, like PatchTST [21], offer tools that can be applied across multiple time-series problems, which reflects a growing trend toward general-purpose models. These overlaps suggest that solutions developed for one task can often be adapted to others, especially when similar model architectures or representations are used.

## 3. Models

A fundamental objective of this research is to conduct a systematic review of different classification models applied to time-series analysis. The study primarily examines two overarching model categories:

- Naive models
- Feature- and pattern-based models.

To ensure a meaningful comparison, a diverse set of accuracy metrics is employed, providing a structured assessment of model performance.

### 3.1. Naive Methods

In this research, two naive time-series classification methods were implemented. Both of them assign class labels to each of the class randomly. For all further explanations, we adopt the following notation:

- *c* denotes the class label,
- *j* is the class index (iterator), where 1≤j≤k,
- *k* represents the total number of classes,
- $n_{j}$ indicates the number of occurrences of class $c_{j}$ in the dataset,
- *N* denotes the total number of observations,
- *p* indicates the number of time series (columns in dataset).

The first of the introduced approaches works on the basis of assigning labels according to a uniform distribution, meaning that each class has an equal probability of being selected, regardless of its actual prevalence in the dataset. The probability of assigning a class label $c_{j}$ is given by

(1) $$P\left(c_{j}\right)=\frac{1}{k},$$

where 1≤j≤k and *k* is the total number of classes.

The second method, referred to as the *weighted method*, assigns classes following the weighted probability distribution, where the likelihood of a class being assigned corresponds to its relative frequency in the dataset.

(2) $$P\left(c_{j}\right)=\frac{n_{j}}{N}$$

This method makes it more likely to assign classes which occur more frequently, matching the distribution of the dataset. If class distributions are balanced, both approaches yield equivalent results, as shown below:

(3) $$n_{j}=\frac{N}{k}\;\Rightarrow \;P\left(c_{j}\right)=\frac{n_{j}}{N}=\frac{\frac{N}{k}}{N}=\frac{1}{k}.$$

The weighted method can be particularly useful for datasets in which class imbalance exists, as it increases the likelihood of assigning underrepresented classes according to their actual occurrence, thus avoiding biases caused by uniform random assignment.

### 3.2. Used Models

Throughout the years, a variety of time-series classification algorithms have been proposed, each of them applying distinct theoretical methodologies. To maintain a diverse range of analytical approaches, we selected a subset of models which includes: convolutional, dictionary-based, feature-extraction, deep learning, and interval-based techniques. Each category presents a diverse approach to processing time-series data, focusing on different aspects such as convolutional transformations, symbolic representations, feature extraction, or interval-based analysis. For this study, representative algorithms from each of the above-mentioned categories were chosen to combine a wide perspective with a concise review. These include the Rocket classifier (convolution-based) [7], the WEASEL (dictionary-based) [22], the Catch22 [8] and the TSFresh (feature-based) [23], the CNN Classifier (deep learning) [6] and the TSF (interval-based) [24]. The following sections describe these algorithms in detail, pointing out their unique features and contributions to time-series classification.

The ROCKET (RandOm Convolutional KErnel Transform) method is part of the convolution-based group and is well known for its efficiency and effectiveness [25]. This classification technique processes data using convolutional kernels, which are randomly generated and this randomness applies to parameters such as weight, length, direction, and bias. The ROCKET method generates a large set of random kernels, which are applied to time-series data in order to extract the most informative features. The following equation illustrates the convolution operation used in the approach. It shows how a kernel, with a specified dilation, is applied to a segment of the input time series to produce a feature map.

(4) $$X_{i}*\omega =\sum_{j=0}^{l_{\mathrm{kernel}}-1}X_{i+(j\times d)}\cdot \omega_{j}$$

where $X_{i}$ is the input time-series segment, ω is the kernel, and *d* is the dilation parameter [7]. This process enables the ROCKET algorithm to efficiently capture both local and global patterns in time-series data, ensuring that it remains effective for a wide range of classification tasks.

The WEASEL (Word Extraction for Time Series Classification) algorithm is a dictionary-based model. This classifier expands the standard dictionary-based approach (e.g., the BOSS algorithm [26]) by focusing on significant patterns and relationships in the data [22]. It takes into account the sequence of symbols and selects only the most relevant features for classification. It extracts normalized windows of varying lengths from the time series, transforms them into symbolic words (called unigrams and bigrams), and encodes them in a discriminative bag-of-patterns representation. This representation captures both individual patterns and relationships between neighboring patterns. The Chi-Squared test is applied to filter irrelevant words, ensuring that only the most relevant features are selected for classification [27].

Both Catch22 and TSFresh classifiers are feature-based algorithms. The Catch22 (CAnonical Time series CHaracteristics) Classifier utilizes 22 key features to capture the dynamics of time-series data [8]. The 22 features in Catch22 were carefully chosen to balance computational efficiency and classification performance. This set includes diverse characteristics of time series, such as metrics that describe autocorrelation patterns (both linear and non-linear), changes in value across time steps, and statistical properties related to the distribution of values, such as outliers. It was developed to address the issue of high computational cost in time-series classification. Earlier feature-based methods employed a significantly larger set of features, whereas Catch22 focuses on a compact selection of features that retain strong classification capability [28]. The TSFresh (Time Series FeatuRe Extraction on basis of Scalable Hypothesis tests) classifier operates by automatically extracting and selecting features from time-series data [23]. This algorithm uses 63 characterization methods to compute a comprehensive set of 794 features that broadly describe the data’s structure. The generated features are then subjected to statistical hypothesis testing to identify the most relevant ones. This approach allows for the extraction of complex, yet meaningful, patterns within the data, improving both interpretability and classification performance.

CNN (Convolutional Neural Network) Classifier is a deep-learning technique originally developed for object recognition and later adapted for time-series classification. It automatically discovers and extracts the internal structure of time-series data to generate deep features for classification. Convolution and pooling operations alternate to produce these features from raw input, and their output is fed into a multilayer perceptron that performs the final classification. This approach was designed to improve robustness to high noise levels. One limitation is that the time-series length must remain fixed during both training and testing, which can be challenging with real-world data [6].

Interval-based classifiers operate by dividing a time series into multiple intervals and extracting key features from each segment. The TSF (Time Series Forest) Classifier is one such interval-based algorithm, involving the random selection of multiple intervals and the calculation of statistics for each. One of the distinguishing features of the TSF is the use of the entrance gain, which combines information about entropy and distance to select optimal splits at each stage of the decision tree more effectively. This method improves the classifier’s ability to separate different classes in time-series data. By relying on simple statistics, such as mean, standard deviation, and slope, the TSF remains computationally efficient, with a runtime that scales linearly with the length of the time series. In the TSF, each interval is processed independently through decision trees within a forest classifier, where each interval functions as a separate entity. The final classification decision is made through a voting process based on the results from these trees [24].

### 3.3. Accuracy Measures

In the field of data classification, numerous accuracy metrics have been proposed, all of which can be applied to classification models irrespective of whether the input data include time series. A holistic approach is essential for obtaining a performance evaluation of the built models using various data types. Therefore, rather than relying on a single metric, a whole set of metrics is necessary to accurately reflect the model’s efficiency.

Based on the general concept of model efficiency measurement, five fundamental effectiveness indicators are commonly used: accuracy, precision, F1, recall and AUC [29]. Analyzing these metrics together provides a comprehensive insight into model performance. The accuracy metric calculates the ratio of correctly predicted instances to all the instances, which were taken into account. The precision, on the other hand, measures the ratio of true positive predictions to the sum of true positives and false positives, indicating how many of the positive predictions are actually correct. The recall is the ratio of true positives to all actual positive instances, showing the model’s ability to identify positive cases. The F1 score combines precision and recall by calculating their harmonic mean, offering a balanced metric when both are crucial [30]. Finally, the AUC (Area Under the Curve) represents the area under the ROC curve, providing insight into the model’s ability to distinguish between classes [31].

Adding additional extra metrics, which are derived from general accuracy measures concept: balanced accuracy and average precision, can address specific dataset characteristics. The balanced accuracy is calculated as the average recall (sensitivity) across all classes, making it particularly useful for imbalanced datasets [32]. The average precision represents the mean precision across different threshold values, offering a thorough examination of the model’s performance at varying levels of recall [33].

Apart from the most common indicators and their variations, there are also Jaccard Metric (a measure of set similarity, particularly useful in multilabel classification) [34], Cohen’s Kappa (a metric that accounts for chance agreement, used to assess classifier consistency [35]), and Matthews Correlation Coefficient (a coefficient that considers all values of the confusion matrix and is regarded as an indicator of overall classifier accuracy) [36], which are worth considering when trying to understand full perspective of model performance.

## 4. Data Simulations

This study focuses on artificially generated time series data. To investigate the full scope of performance for classification models, 82 datasets were generated. The generated datasets consist of multiple time series and, to ensure full representativeness, they differ from each other in the number of observations, time series lengths, wave types, and noise distribution. Each individual time series was generated from one of the predefined shapes (also referred to as the wave types) and, if specified, with added noise.

### 4.1. Wave Types

**Figure 1:**
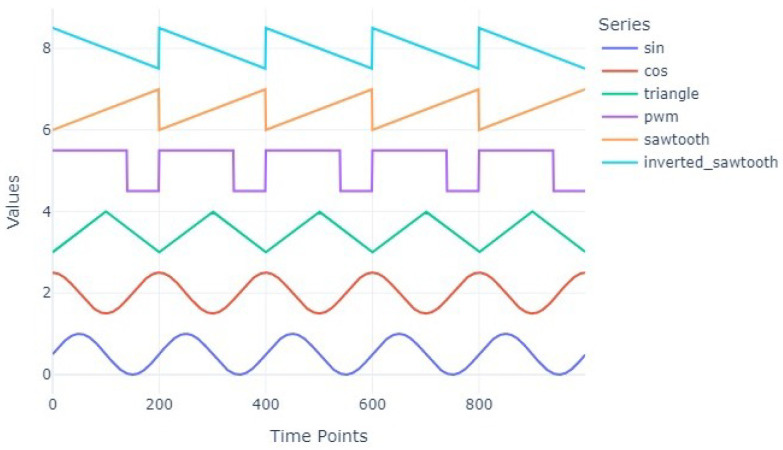
All shapes time series.

Seven functions were selected as shapes creating data points for time series: sin, cos, square, triangle, sawtooth, inverted sawtooth and pwm. Each wave type was defined by three characteristics: minimum value, maximum value and frequency. The minimum and maximum values represent lower and upper boundaries of the signal values. The frequency, on the other side, specifies the number of cycles generated across the entire length of time series. Figure 1 presents all the wave types.

For each time series shape, the following parameters are used:

- *t*—time index; t=0,1,…,length−1;
- freq—frequency of the wave, determining how many cycles occur across the series length;
- min—minimum value of the amplitude;
- max—maximum value of the amplitude;

The mathematical formulas for different time series shapes and their parameters are given below:

1. Sinusoidal Wave (‘sin’)
  The sinusoidal wave is defined by:
  (5) $$y\left(t\right)=\frac{\max-\min}{2}\cdot \sin\left(2\pi \cdot \mathrm{freq}\cdot \frac{t}{\mathrm{length}}\right)+\frac{\max+\min}{2},$$
  - The term $\frac{\max-\min}{2}$ represents the amplitude of the wave.
  - The offset $\frac{\max+\min}{2}$ centers the wave between min and max.
2. Cosinusoidal Wave (‘cos’)
  The cosinusoidal wave is defined by:
  (6) $$y\left(t\right)=\frac{\max-\min}{2}\cdot \cos\left(2\pi \cdot \mathrm{freq}\cdot \frac{t}{\mathrm{length}}\right)+\frac{\max+\min}{2},$$
  - The formula is similar to the sinusoidal wave, but it starts at its maximum value when t=0.
3. Triangle Wave (‘triangle’)
  The triangle wave is defined by:
  (7) $$y\left(t\right)=\frac{\max-\min}{2}\cdot \mathrm{sawtooth}\left(2\pi \cdot \mathrm{freq}\cdot \frac{t}{\mathrm{length}},0.5\right)+\frac{\max+\min}{2},$$
  - The sawtooth(x,0.5) function generates a triangular waveform with symmetrical peaks and valleys.
4. Sawtooth Wave (‘sawtooth’)
  The sawtooth wave is defined by:
  (8) $$y\left(t\right)=\frac{\max-\min}{2}\cdot \mathrm{sawtooth}\left(2\pi \cdot \mathrm{freq}\cdot \frac{t}{\mathrm{length}}\right)+\frac{\max+\min}{2},$$
  - This wave rises linearly to its peak and then drops sharply to the minimum value.
5. Inverted Sawtooth Wave (‘inverted_sawtooth’)
  The inverted sawtooth wave is defined by:
  (9) $$y\left(t\right)=-\frac{\max-\min}{2}\cdot \mathrm{sawtooth}\left(2\pi \cdot \mathrm{freq}\cdot \frac{t}{\mathrm{length}}\right)+\frac{\max+\min}{2},$$
  - This wave descends linearly to the minimum value and then jumps sharply to the maximum.
6. Pulse Width Modulation (PWM) Wave (‘pwm’)
  The PWM wave is defined by:
  (10) $$y\left(t\right)=\frac{\max-\min}{2}\cdot \mathrm{sgn}\left(\sin\left(2\pi \cdot \mathrm{freq}\cdot \frac{t}{\mathrm{length}}\right)-\mathrm{duty}\right)+\frac{\max+\min}{2},$$
  - The duty parameter determines how long the signal stays “high” during each cycle.
  - A duty cycle of 0.5 results in equal time spent in the “high” and “low” states.
  - By default, in our study, the duty parameter is set to 0.7, meaning that the signal remains “high” for 70% of each cycle.

**Figure 2:**
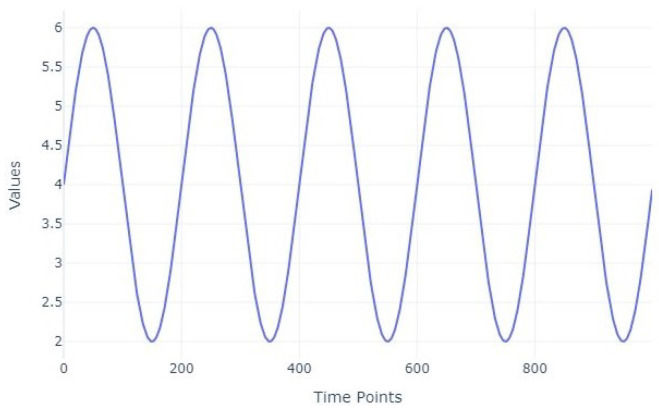
Time series with sinusoidal shape.

An example of a time series of length 1000 generated from a sinusoidal function with a minimum value of 2, a maximum value of 6, and a frequency of 5 is shown in Figure 2.

### 4.2. Noise

Once each time series has been generated using the predefined function, a noise value from one of the distributions was added to the time series value. This was done in order to create a more realistic datasets and evaluate the real classification performance of the examined models.

In this study, the noise was drawn from a normal distribution characterized by its mean and standard deviation. For the purposes of the experiment, the mean was set to 0 for all the cases, while for standard deviation different values were tested as data fluctuations. The main aim of these modifications was to assess their impact on model performance.

**Figure 3:**
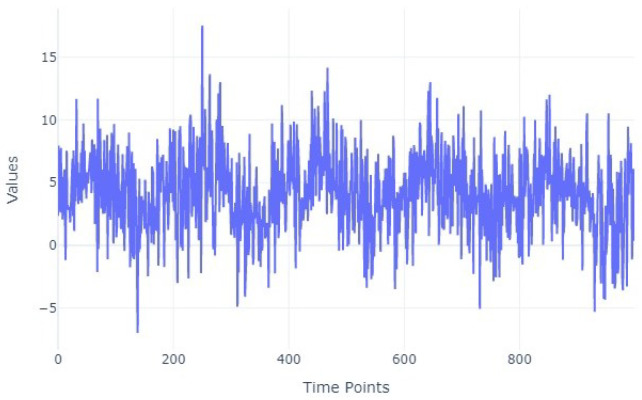
Time series with sinusoidal shape and noise.

Figure 3 illustrates a sinusoidal time series generated with the same parameters as in Figure 2 (minimum value = 2, maximum value = 6, and frequency = 5); however, noise was added. The noise was drawn from a normal distribution with mean value 0 and a standard deviation equal to 3, demonstrating the effect of noise on the original signal.

### 4.3. Datasets

As mentioned above, within this research project, 82 datasets were generated. These datasets differ in the number of classes, which ranged from 2 to 6, and the distribution that the noise was generated from. The datasets are categorized as follows:

1. 10 datasets with two classes: cosinusoidal and PWM.
2. 18 datasets with three classes: cosinusoidal, PWM, and sinusoidal.
3. 18 datasets with four classes: cosinusoidal, PWM, sinusoidal, and triangular.
4. 18 datasets with five classes: cosinusoidal, PWM, sinusoidal, triangular, and sawtooth.
5. 18 datasets with six classes: cosinusoidal, PWM, sinusoidal, triangular, sawtooth, and inverted sawtooth.

The above shape combinations were chosen to ensure the maximum diversity within the each dataset. Across each class configuration, the datasets differ only in the standard deviation of the noise added to the time series. This assures that variations within a group highlight the effect of noise intensity on a model performance, while the fundamental class structure and waveform characteristics remain stable.

Each dataset comprises of an equal number of samples for all classes, ensuring balanced class distributions. Each dataset contains 200 time series, with 1000 observations per series. All of the waveforms had the same input parameters as follows: a minimum value of 2, a maximum value of 6, and a frequency value equal to 8. Moreover, no missing values were introduced, as the primary objective of this study is to assess the performance of classification models under varying noise levels, rather than their robustness to incomplete data.

The noise was sampled from a normal distribution with a mean value equal to 0. For datasets containing three or more classes, the standard deviation of the noise ranged from 1 to 9 (with increments of 1). For all datasets, additional standard deviation values ranged from 5 to 50 (with increments of 5). This experimental design offers an extensive framework for evaluating the performance of the models under different levels of noise and varying degrees of complexity.

## 5. Experiment

This section presents the results obtained from the conducted experiments. Seven classification models were trained for each dataset: Naive Classifier, Rocket Classifier, WEASEL, Catch22 Classifier, TSFresh Classifier, Time Series Forest Classifier, and CNN Classifier. To ensure a robust evaluation, we employed Stratified K-Fold cross-validation with 10 folds. Each dataset was divided into 10 subsets, with the model iteratively trained on 9 folds while the remaining fold was used for validation. This approach ensured that every data point contributed to both training and validation, providing a reliable and unbiased estimation of model performance. All the models were implemented using the *aeon* package in Python (https://www.python.org/) [5]. For each model, we experimented with different sets of hyperparameters:

- Catch22 Classifier: n_estimators = 50, 100, 250, 500;
- Rocket Classifier: n_kernels = 100, 1000, 5000, 10,000;
- Time Series Forest Classifier: n_estimators = 50, 100, 250, 500;
- TSFresh Classifier: default_fc_parameters = “efficient”, “minimal”;
- WEASEL Classifier: window_incint = 2, 4, 8; alphabet_size = 2, 4, 8;
- CNN Classifier: n_epochs = 100, 1000, 2000;

All the other parameters were set to their default values. Having trained each model, we selected the best-performing configurations based on the evaluation accuracy metric calculated on the test set. The best-performing models were selected at the fold level. When multiple parameter combinations yielded the same accuracy, the choice was made based on training time to maximize overall efficiency, taking into account both predictive performance and computational cost. The final model parameters were chosen to ensure the best adaptation to the data.

### 5.1. Computational Resources

All experiments were run on the “Topola” high-performance computing (HPC) cluster at the Interdisciplinary Centre for Mathematical and Computational Modelling (ICM), University of Warsaw. The Topola cluster provides 64 nodes compute, each equipped with dual Intel Xeon Gold 6230 CPUs, 192 GiB RAM per node, GPUs: 8 NVIDIA Tesla V100 GPUs (32 GiB HBM2 each) available on four GPU-enabled nodes.

### 5.2. Results

Naive models were constructed as the baseline classifiers to serve as reference points for evaluating the performance of more complex algorithms. Through an analytical comparison of advanced models to the naive ones, it is possible to assess whether performance improvements are significant or if no clear pattern can be noticed. In the case of balanced classes, such models help to highlight whether a classifier utilizes meaningful patterns or performs close to random guessing. Since these models assign classes randomly, their performance depends only on the number of classes and does not take into account the level of data perturbation such as the noise incorporation.

The consistency of mean values for each metric in the Naive Classifier within groups of datasets with the same number of classes occurs from its random prediction process. In each group, datasets contain 200 time series, evenly distributed among the classes. As a result, regardless of the noise standard deviation or any other parameters, the random assignment of predictions yields the same average performance within each group, but different values across groups with varying numbers of classes.

When a fewer number of classes was considered, the models exhibited higher performance metrics, particularly when low noise conditions were considered. This clearly indicates greater robustness in simpler classification tasks, i.e., when the number of classes is limited. Even when the noise level increased, the models were more resilient in maintaining their performance due to the reduced complexity of classifying between a smaller set of classes. However, as the number of classes increased, the classification task became more challenging, resulting in a significant decrease in a model’s performance. Overall, to sustain a comparable level of model performance as the number of classes increases, it is necessary to limit the noise introduced into the data by reducing its standard deviation.

Different measures were used to assess models’ performance; however, accuracy was chosen as the primary measure. One of the main reasons was the balanced distribution of classes across all datasets. Since the data were synthetically generated, each dataset contained the exact same number of observations per class. In this case, accuracy provides a reliable and interpretable assessment of a model’s predictive capability, as it equally reflects the proportion of correct predictions without being biased towards any specific class.

**Figure 4:**
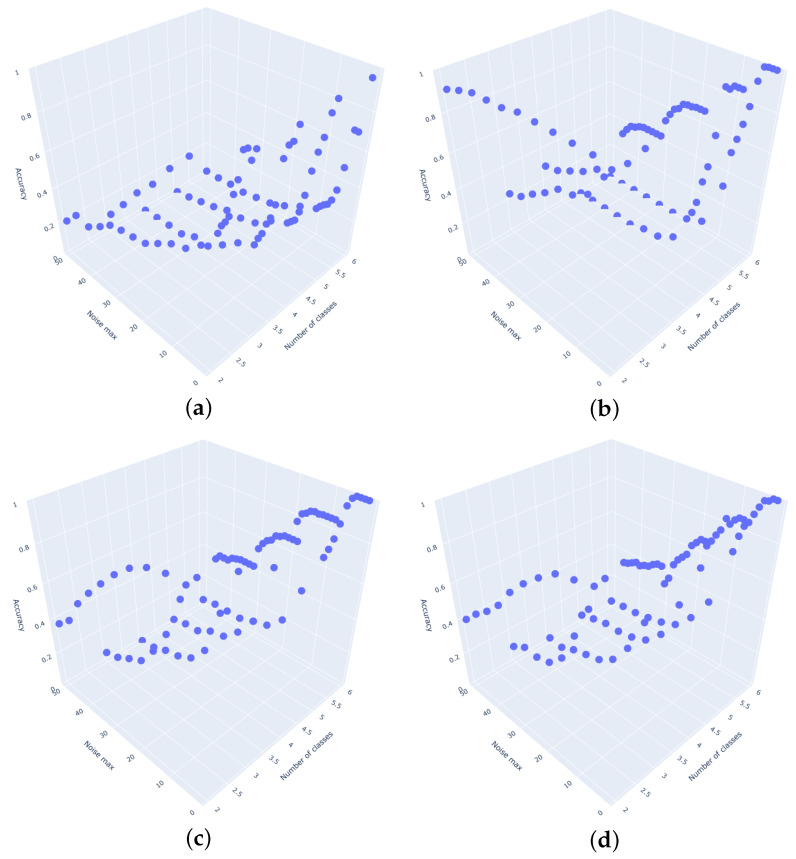

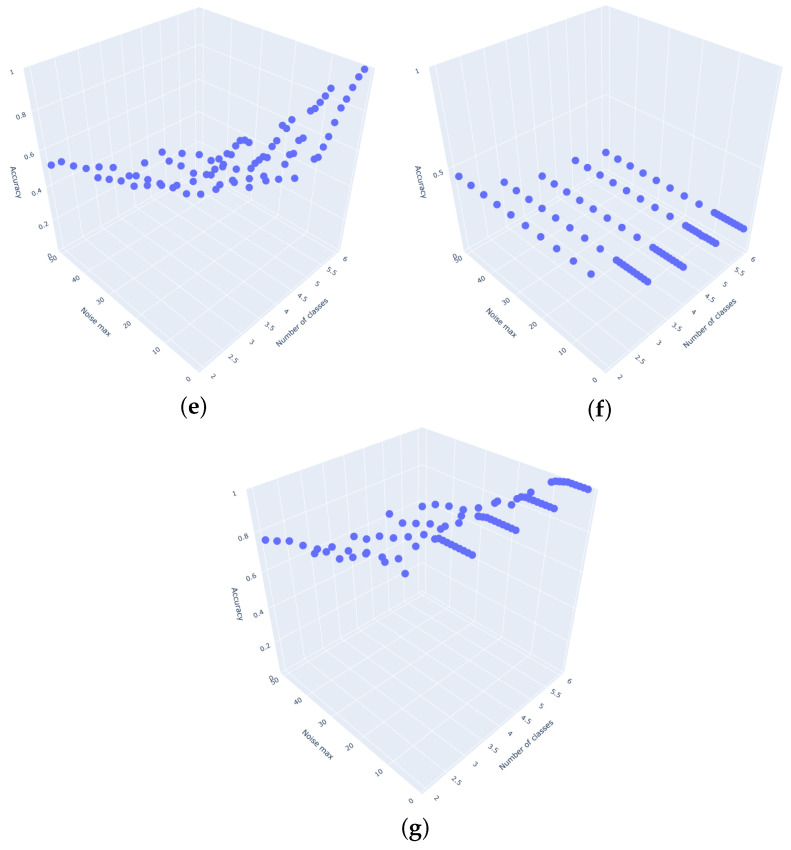
Classifier accuracy for different number of classes and noise standard deviation. (**a**) Catch22 Classifier. (**b**) Rocket Classifier. (**c**) Time Series Forest Classifier. (**d**) TSFresh Classifier. (**e**) WEASEL Classifier. (**f**) Naive Classifier. (**g**) CNN Classifier.

**Figure 5:**
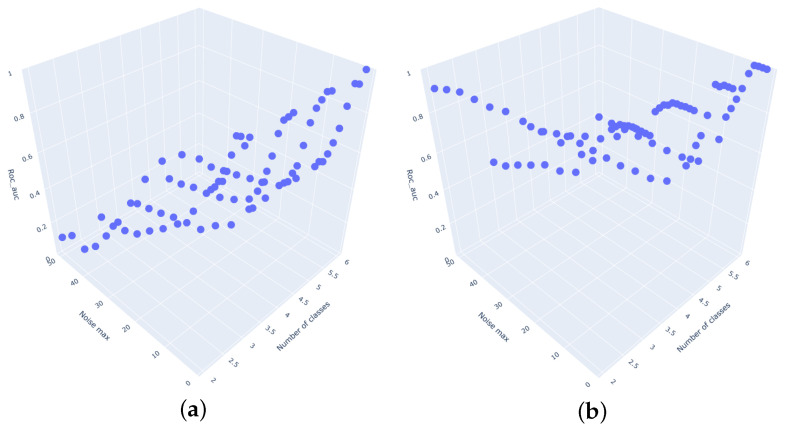

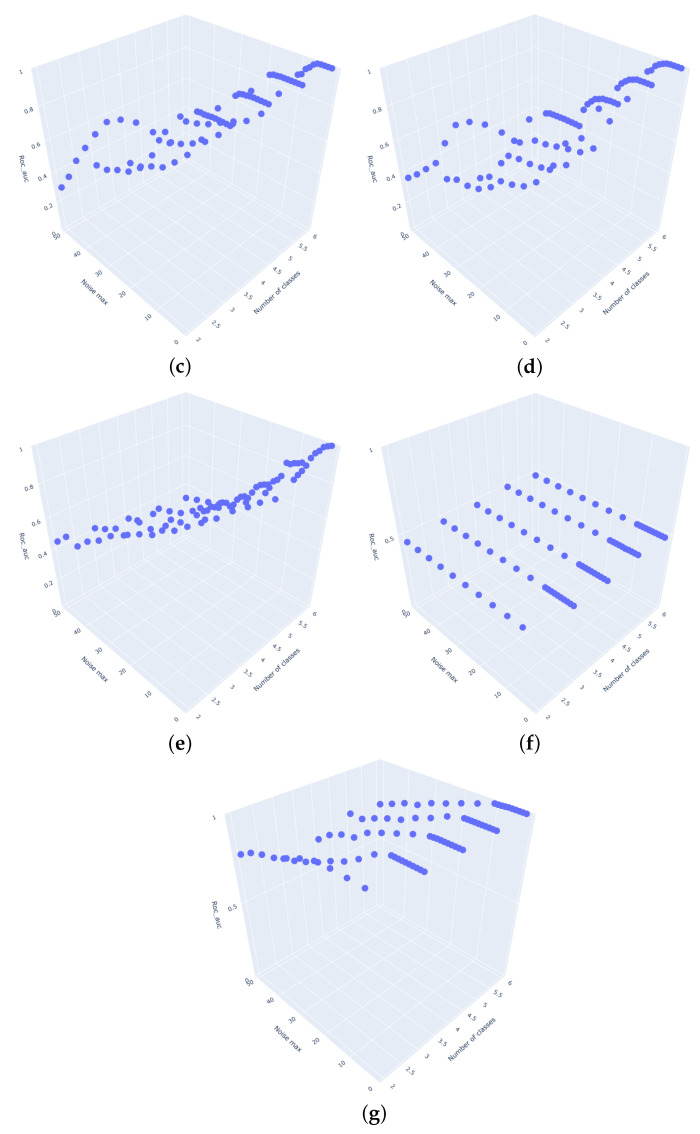
Classifier AUC for different number of classes and noise standard deviation. (**a**) Catch22 Classifier. (**b**) Rocket Classifier. (**c**) Time Series Forest Classifier. (**d**) TSFresh Classifier. (**e**) WEASEL Classifier. (**f**) Naive Classifier. (**g**) CNN Classifier.

Figure 4 presents the average accuracies of the classifiers for the testing datasets, grouped by the standard deviation of the noise and the number of classes. Similarly, Figure 5 illustrates the average AUC values for the same datasets. All the above-mentioned plots were generated in 3D and can also be accessed here (https://github.com/MariaSadowska/TSC_classification_article.git) (accessed on 10 March 2025).

**Figure 6:**
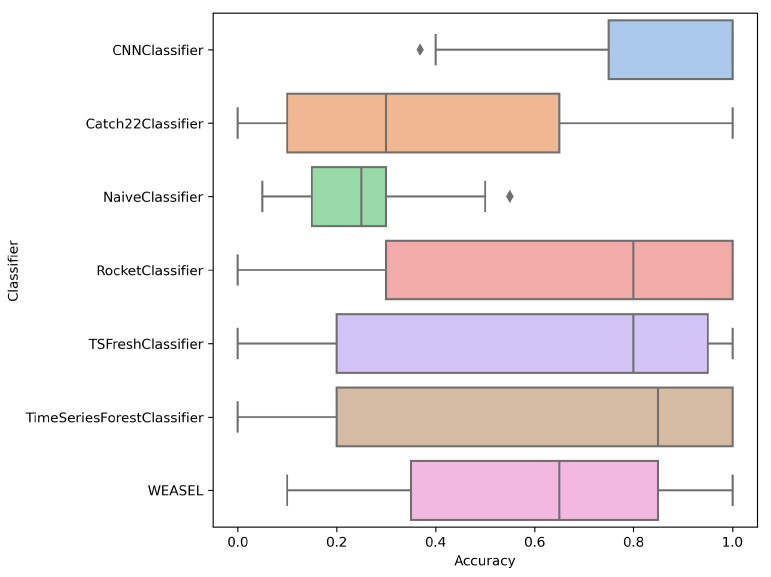
Accuracy distribution by classifiers.

Figure 6 presents a boxplot of the accuracy metric for all the classifiers. It is clearly shown that the highest median value was achieved by the CNN Classifier. Time Series Forest Classifier, Rocket Classifier and TS Fresh Classifier exhibit similarly high median values, albeit slightly lower. This indicates that the aforementioned classifiers are stable and effective models.

Table 1 presents the results of Dunn’s test for each classifier, considering α=0.05. Dunn’s test is a post-hoc analysis used to assess the significance of statistical differences between groups. Classifiers sharing the same letter do not differ significantly.

The CNN classifier is labeled ‘a’, indicating the best performance, and no other model shares its group, so its results differ significantly from all others. Time Series Forest, Rocket, TSFresh, and WEASEL classifiers are all marked with ‘b’, indicating their results are not significantly different from each other. Catch22 and Naive classifiers both carry the label ‘c’, meaning their performances do not differ significantly from each other, and, per Dunn’s test, Catch22 classifier outcomes are indistinguishable from random guessing.

**Figure 7:**
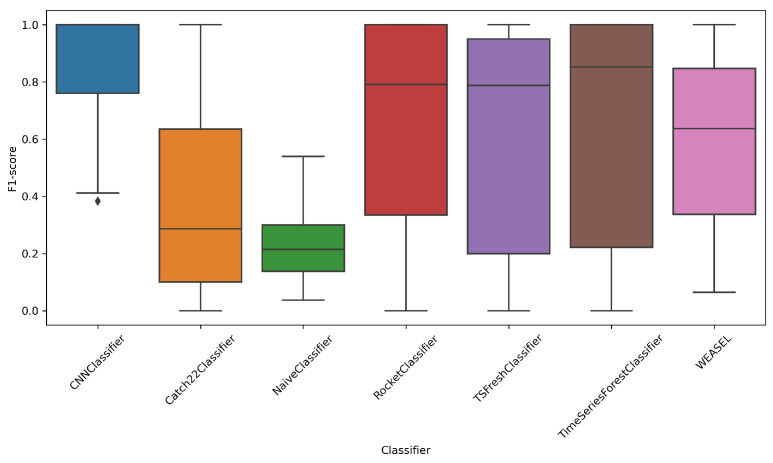
F1 distribution by classifiers.

Figure 7 presents the boxplot of the F1 score metric for each classifier. The results are consistent with the accuracy boxplot, indicating that the CNN Classifier demonstrates the highest robustness to the number of classes and noise levels. Median values for Catch22 and Naive classifiers are similarly low.

Training times are compared across classifiers for each combination of class count and noise level. Multiple hyperparameter settings were tested per scenario, and we chose the setting with the highest test accuracy, additionally when several settings matched, the fastest one was selected. As a result, exactly one hyperparameter combination is reported for each classifier under each (classes, noise) condition.

**Figure 8:**
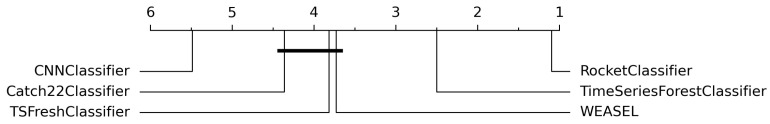
Comparison of classification algorithms’ training times on generated datasets.

**Figure 9:**
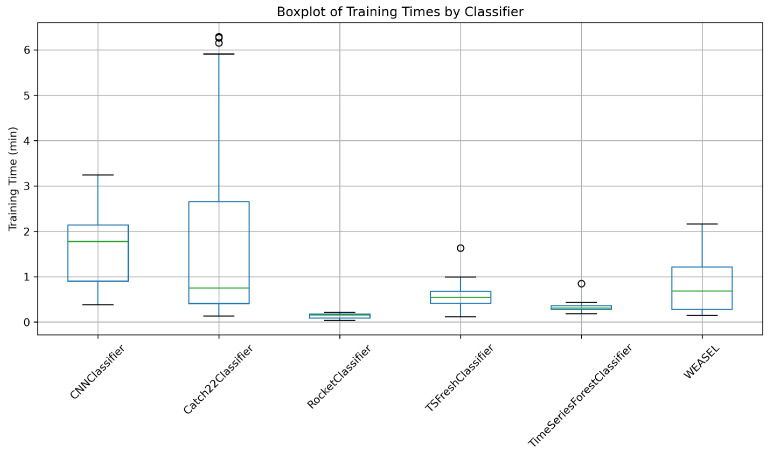
Distribution of training times by classifiers.

Figure 8 presents the critical difference (CD) diagram, illustrating the results of the rank analysis of classifiers based on their training time, assuming α=0.05. The diagram presents Rocket Classifier as the most effective one with the shortest training time and CNN Classifier as the model with longest time. This diagram indicates that the WEASEL, TSFresh and Catch22 classifiers’ training time differences are not statistically significant. Similar insights emerge from the training-time distributions shown in Figure 9. The Rocket Classifier exhibits the narrowest box and the lowest median. Among all models, the CNN Classifier stands out with a substantially larger training time—its median exceeds 1.77 min, whereas the medians of all other classifiers remain below 0.75 min.

Table 2 reports the observed peak memory usage (in MiB) for a representative run of each time-series classification model under its selected hyperparameter configuration. Notably, the Catch22 classifier has the lowest memory footprint, despite its performance being statistically indistinguishable from random guessing. The WEASEL classifier demands the most memory—approximately 409% more than the CNN, which achieves the best results—yet its accuracy does not surpass that of the top-performing models.

All of the above analyses focused on comparing model performance. The Naive Classifier exhibited the lowest efficiency, which was an expected outcome, as its primary purpose was to establish baseline metric values for random class assignment. By comparing other models to the Naive Classifier, we were able to assess their actual performance. The results of all conducted analyses indicate that Rocket Classifier, Time Series Forest Classifier, and TS Fresh Classifier achieve similar efficiency, while the Catch22 Classifier demonstrates the lowest performance. CNN Classifier presents the highest efficiency across all built models.

The following part of the analysis aims at studying the influence of input data type on the results of the classifiers. Thus, noise levels 1 to 4 and 6 to 9 were excluded from the analysis, as datasets with two classes included noise values ranging only from 5 to 50 (in increments of 5). Including these lower noise levels selectively could cause inconsistencies in the comparison, as models trained on two-class datasets typically demonstrated enhanced efficiency, and that could have caused bias in the results and might have disturbed our conclusions.

**Figure 10:**
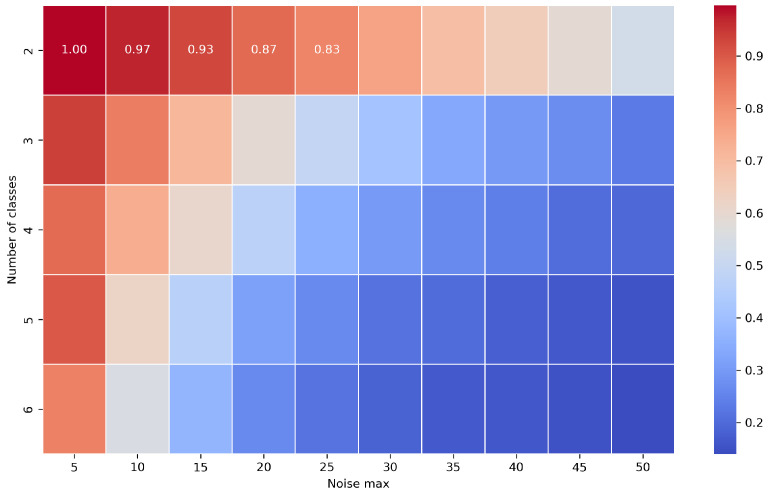
Accuracy heatmap across noise levels and number of classes (excluding Naive Classifier).

Figure 10 presents a heatmap of the mean classification accuracies across various noise levels and numbers of classes. The results are averaged across all classification models used in this study, excluding the Naive Classifier. These models include Catch22, Time Series Forest, TSFresh, CNN, ROCKET, and WEASEL. Accuracy was computed as the average proportion of correct predictions on the test set for each combination of noise level and number of classes. The results clearly show that the highest performance was achieved with a small number of classes and minimal data disruptions. Models trained on two-class problems exhibited greater robustness to noise compared to models trained on a larger number of classes.

Table 3 and Table 4 present the models’ performance across different numbers of classes (mean values) and varying noise standard deviations—excluding noise values from 1 to 4 and 6 to 9.

Table 3 presents the mean accuracy results compared across different number of classes. CNN Classifier demonstrated the highest overall performance for most of the classes configurations, whereas Catch22 Classifier recorded the lowest mean accuracy. There was a little difference between the Rocket, TSFresh, WEASEL, and Time Series Forest classifiers. Although the general analysis indicated that the CNN Classifier was the best performing model, for the two-classes datasets it was Rocket Classifier which achieved the highest accuracy. However, its accuracy radically drops as the number of classes increases. A noticeable decline in model performance was observed as the number of classes increased. An analysis of mean accuracy values and standard deviations across all the classifiers indicates a similar response to datasets with varying class counts. Notably, CNN Classifier exhibited the lowest performance variability, as reflected by its standard deviation.

Table 4 presents the results of model accuracies across varying noise levels. A comparison of model performance across different noise standard deviations suggests that TSF classifiers exhibited the lowest resilience to increasing noise levels.

For all the classifiers, higher levels of data perturbation and more number of classes resulted in a noticeable decline in the performance. The detailed results are available in the Excel file on the public GitHub repository at the following link: https://github.com/MariaSadowska/TSC_classification_article.git (accessed on 10 March 2025).

### 5.3. Validity Threats

Overall, it can be concluded that there are no risks concerning the validity assessment of the results obtained in our research. The entire study was based on numerical simulations conducted using Python, a widely accessible and free programming language. As mentioned previously, the source code for the implemented *TSC* package is publicly available on GitHub (https://github.com/), specifically to facilitate validation and error reporting. No external data were utilized, as all simulations were carried out under controlled conditions, ensuring the preservation of the seed for pseudo-random number generators. This guarantees that any researcher can fully replicate our empirical study with 100% accuracy. To reveal underlying dependencies, our findings have been presented in both tabular and graphical formats. However, understanding the core principles of the proposed approach and interpreting the results may depend on the researcher’s familiarity with computer technologies, statistical and evolutionary algorithms, as well as their proficiency in the given programming language.

## 6. Conclusions

This study aimed to analyze the efficiency of the time-series classification models based on the input data characteristics. The results presented demonstrate the influence of the number of classes and noise levels on algorithm performance. The experiment was conducted in two main stages: first, the generation of synthetic time-series datasets; and second, the construction and evaluation of classification models. The findings indicate that the CNN Classifier demonstrated strong effectiveness, while the Catch22 Classifier produced the least accurate classifications among the evaluated models. Additionally, the results revealed a clear relation between the input data characteristics and the classification efficiency. Specifically, model performance declined with an increase in the number of classes, as well as with the higher noise levels.

This study contributes to the field by providing a comprehensive comparison of time-series classification models under varying input conditions and offering insights into how data characteristics influence model performance. Future research could focus on additional factors affecting classification performance, such as the length of time-series data, the number of observations, and the impact of imbalanced data on model efficiency. These aspects could provide a more in-depth understanding of how time-series classification models could be optimized for different applications.

## Figures and Tables

**Table 1:** Dunn’s test results for comparison of classifiers.

Classifier	Measure	Group
CNN Classifier	0.8812	a
Time Series Forest Classifier	0.6493	b
Rocket Classifier	0.6380	b
TSFresh Classifier	0.6176	b
WEASEL	0.6056	b
Catch22 Classifier	0.3794	c
Naive Classifier	0.2483	c

**Table 2:** Peak memory usage of time-series classification models (in MiB).

Classifier	Peak Memory (MiB)
Catch22	10.94
Time Series Forest	17.10
TSFresh	66.59
CNN	72.02
Rocket	101.30
WEASEL	367.38

**Table 3:** Mean accuracy of classifiers for different numbers of classes on test sets.

d	CNN	Catch22	Rocket	TSFresh	TSF	WEASEL
2	0.9035	0.5560	**0.9785**	0.7455	0.7670	0.7410
3	**0.7881**	0.2509	0.5495	0.4678	0.4713	0.5462
4	**0.7890**	0.1260	0.5615	0.2920	0.3645	0.4180
5	**0.7805**	0.1435	0.1478	0.3032	0.3492	0.3637
6	**0.7686**	0.1015	0.1348	0.2661	0.2756	0.2837
μ	**0.8060**	0.2356	0.4744	0.4149	0.4455	0.4705
σ	0.0493	0.1682	0.3128	0.1799	0.1725	0.1600

**Table 4:** Mean accuracy of classifiers for selected noise levels.

Noise	CNN	Catch22	Rocket	TSFresh	TSF	WEASEL
5	**1.0000**	0.6461	0.9806	0.9602	0.9932	0.8665
10	**0.9869**	0.4062	0.6925	0.8114	0.8894	0.6774
15	**0.9328**	0.3311	0.5136	0.6233	0.7346	0.5609
20	**0.8545**	0.2577	0.4433	0.4589	0.5298	0.4768
25	**0.8173**	0.2025	0.4122	0.3444	0.3741	0.4387
30	**0.7631**	0.1655	0.3901	0.2649	0.2850	0.3833
35	**0.7220**	0.1112	0.3629	0.2137	0.2247	0.3643
40	**0.6918**	0.0923	0.3409	0.1845	0.1816	0.3371
45	**0.6598**	0.0852	0.3127	0.1595	0.1355	0.3150
50	**0.6313**	0.0582	0.2956	0.1285	0.1073	0.2850
μ	**0.8060**	0.2671	0.4744	0.4149	0.4455	0.4705
σ	0.1276	0.1742	0.2014	0.2772	0.3078	0.1744

## Data Availability

Data available in a publicly accessible repository: GitHub https://github.com/MariaSadowska/TSC_classification_article, accessed on 1 February 2025.
